# IGF‐1 Signaling Plays an Important Role in the Formation of Three‐Dimensional Laminated Neural Retina and Other Ocular Structures From Human Embryonic Stem Cells

**DOI:** 10.1002/stem.2023

**Published:** 2015-05-13

**Authors:** Carla B. Mellough, Joseph Collin, Mahmoud Khazim, Kathryn White, Evelyne Sernagor, David H. W. Steel, Majlinda Lako

**Affiliations:** ^1^ Institute of Genetic MedicineNewcastle UniversityNewcastleUnited Kingdom; ^2^ Institute of NeuroscienceNewcastle UniversityNewcastleUnited Kingdom; ^3^ EM Research Services, Newcastle UniversityNewcastleUnited Kingdom; ^4^ Sunderland Eye InfirmarySunderlandUnited Kingdom

**Keywords:** Neural retina, Retinal pigmented epithelium, IGF‐1, Human pluripotent stem cells, Differentiation

## Abstract

We and others have previously demonstrated that retinal cells can be derived from human embryonic stem cells (hESCs) and induced pluripotent stem cells under defined culture conditions. While both cell types can give rise to retinal derivatives in the absence of inductive cues, this requires extended culture periods and gives lower overall yield. Further understanding of this innate differentiation ability, the identification of key factors that drive the differentiation process, and the development of clinically compatible culture conditions to reproducibly generate functional neural retina is an important goal for clinical cell based therapies. We now report that insulin‐like growth factor 1 (IGF‐1) can orchestrate the formation of three‐dimensional ocular‐like structures from hESCs which, in addition to retinal pigmented epithelium and neural retina, also contain primitive lens and corneal‐like structures. Inhibition of IGF‐1 receptor signaling significantly reduces the formation of optic vesicle and optic cups, while exogenous IGF‐1 treatment enhances the formation of correctly laminated retinal tissue composed of multiple retinal phenotypes that is reminiscent of the developing vertebrate retina. Most importantly, hESC‐derived photoreceptors exhibit advanced maturation features such as the presence of primitive rod‐ and cone‐like photoreceptor inner and outer segments and phototransduction‐related functional responses as early as 6.5 weeks of differentiation, making these derivatives promising candidates for cell replacement studies and in vitro disease modeling. Stem Cells *2015;33:2416–2430*

## Introduction

Many forms of visual impairment and blindness remain incurable. A common route to the loss of sight is the dysfunction or death of the light‐sensitive photoreceptors and underlying supportive retinal pigmented epithelium (RPE). Replenishing lost or damaged cells by cell replacement therapy remains an important strategy to cure patients suffering from blinding forms of outer retinal degeneration characterized by the substantial loss of photoreceptors and RPE. One great advantage for using this approach in the eye is the ease with which de novo cells can be delivered directly to the affected site by subretinal injection. Photoreceptor genesis from a variety of non‐neural cell types from the eye itself (iris pigmented epithelial cells, ciliary epithelium, limbal epithelial cells, and RPE) and bone marrow (hematopoietic and mesenchymal stem cells) is being examined for repopulation of the degenerate retina, so far without notable success 1, 2, 3, 4.

While encouraging photoreceptor integration has been achieved using allogeneic transplantation of primary mouse photoreceptor precursors isolated from the developing neural retina 5, 6, 7, 8, 9, the human equivalent is not a viable resource of transplantable cells and thus such studies in mouse act to provide guiding information on the ideal cellular and host paradigms for optimal integration and functional outcome. Comparable studies using similar stage retinal progenitor cells derived from mouse embryonic stem cells (ESCs) 10, 11, 12 have shown promising results, indicating that pluripotent stem cells could be a suitable resource of transplantable photoreceptors. In the last 10 years, several groups have pursued the differentiation of human pluripotent stem cells, showing that under defined culture conditions, both human ESC (hESC) and human induced pluripotent stem cell (hiPSC) follow a stepwise temporal differentiation process which results in the formation of anterior neural tissue, eye field, and retinal cell generation 13, 14, 15, 16, 17, 18, 19, 20, 21, 22. Key studies have shown that de novo photoreceptor precursors derived from hESCs 23 and hiPSCs 19 can exhibit integrative potential following transplantation into the mouse retina, which has strengthened the basis for the use of pluripotent stem cell‐derived photoreceptors in the human setting. Furthermore, it has been shown that hESC and hiPSC can spontaneously produce various retinal phenotypes in the absence of inductive cues, suggestive of an innate ability that can be exploited to optimize and accelerate the retinal differentiation process 18, 21. Combination of this innate differentiation ability with three‐dimensional culture conditions (3D) has led to generation of optic cup structures with laminated neural retina containing photoreceptor progenitors 22, 24 which, at least in the murine system, could integrate within a degenerate retina and display morphological features of mature photoreceptors 11.

In the human setting, progress toward the clinic is being made with regards to RPE production and replacement from hESC and hiPSC 25, 26, yet achieving the same momentum for photoreceptor replacement has been more difficult. The goal of generating cells in vitro which not only share a similar gene and antigen expression profile but also can elicit a similar electrophysiological profile to native photoreceptors and have the capacity to develop outer segments, necessary for phototransduction, has been met only by one very recent study to date 27. The authors of this study were able to generate laminated retinal tissue from hiPSC containing functional photoreceptors which displayed early outer segment‐like structures in vitro as well as photosensitivity. Yet the protocol used to derive these functional photoreceptors involved many elaborate steps (involving the mechanical dissection of neural retinal domains in two‐dimensional [2D] culture to generate 3D culture), supplementation with different exogenous factors and fetal bovine serum at various stages of differentiation, and took 21 weeks (147 days) to generate a distinct outer nuclear layer and 27 weeks (189 days) for the infrequent observation of functional photoreceptors developing small stacks of outer segment disc‐like structures 27. The complexity of this protocol together with the necessity for manual dissection and the extended timeframe required for the generation of mature photoreceptors makes this approach less suitable for clinical application. With this in mind we set up a screen of mitogens and growth factors which were individually tested under 3D culture conditions in a minimal medium containing the neural supplements B27 and N2, previously shown by our group to play an important role in the differentiation of hESCs toward retinal phenotypes 21. We observed that hESC cultures differentiated with insulin‐like growth factor 1 (IGF‐1), a known enhancer of eye formation and retinal progenitor expression 13, 28, resulted in the formation of numerous optic vesicle‐like structures that matured and formed the bilayered optic cup which gave rise to neural retina and RPE. Furthermore, laminated neural retina formed in the presence of IGF‐1 contained photoreceptors which formed synapses and displayed some key features of mature morphology and membrane attributes compatible with phototransduction by 6.5 weeks of differentiation, indicating an important role for IGF‐1 signaling in both early stages of optic vesicle/cup formation and photoreceptor maturation. Two key studies have shown that it is possible to generate laminated neural retina from hESC and hiPSC under 3D culture conditions using culture media that contained fetal bovine serum and a number of signaling agonists/antagonists and growth factors 24, 27 but not IGF‐1, raising the question of whether IGF‐1 is dispensable for this differentiation process. One needs to note, however, that IGF‐1 is a component of fetal bovine serum 28; hence it is impossible to state that 3D differentiation in these two key studies was achieved in the absence of IGF‐1. Our study was performed in minimal media with neural supplements B27 and N2, thus enabling a simpler assessment of the role of IGF‐1 and its receptors in 3D differentiation of hESC to neural retina in the absence of fetal calf serum which can mask the impact of this factor for the differentiation process.

In addition to the formation of laminated neural retina and RPE, we also observed that addition of IGF‐1 to the medium resulted in the generation of accessory structures such as cornea and lens, a finding which implicates an important role for IGF‐1 in formation of ocular structures from hESC. While the role of IGF‐1 in promoting a retinal fate under 2D culture conditions has been reported by our group 21 and others 13, we believe that this is the first report to highlight an important role for this factor in promoting ocular differentiation from hESC under 3D culture conditions.

## Materials and Methods

### hESC Culture and Differentiation

**Figure 1:**
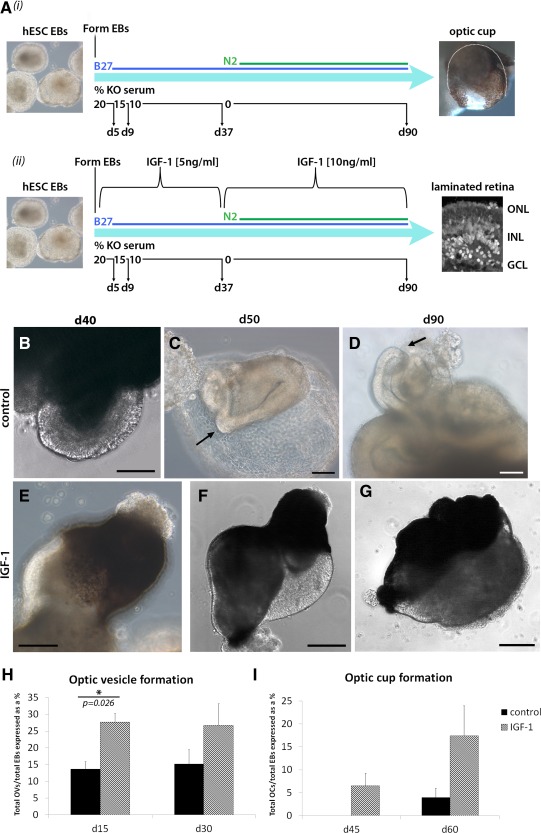
Schematic of our differentiation protocols and examples of the gross morphology of floating hESC‐derived EBs which displayed developmental features associated with neural and retinal development. **(Ai, Aii):** For differentiation, hESCs were grown as EBs in reducing concentrations of KO serum, then as serum‐free cultures from day 37 until day 90. B27 was used as a supplement throughout differentiation, with the addition of N2 supplement from day 37 onwards. (Aii): IGF‐1‐treated cultures were supplemented with 5 ng/ml IGF‐1 for more than days 0–37 and thereafter with 10 ng/ml IGF‐1. **(B–G):** Phase micrographs showing examples of optic vesicular and optic cup structures developing from EBs under control culture conditions (no IGF‐1, B–D) and IGF‐1 treatment (E–G). (C): In some EBs, clear retinal folding could be observed in vitro (arrow indicates hinge). Pictures (E) through (G) show the same EB at days 40, 50, and then 90, demonstrating how complex optic cup structures developed over time. Scale bars: B, D, E = 100 µm, C = 50 µm, F, G = 200 µm. **(H, I):** The treatment of differentiating EBs with IGF‐1 resulted in a significant increase in optic vesicle production by day 15 (H) and higher frequency of optic cup formation (I) when compared with controls. Abbreviations: EB, embryoid body; hESC, human embryonic stem cell; IGF‐1, insulin‐like growth factor 1; KO, knockout.

hESC (line H9, WiCell) was expanded and differentiated as previously described 21; however, EBs were not transferred to attachment culture conditions as done previously, but were maintained in 3D culture throughout differentiation using bacteriological dishes and low attachment six‐well plates (Corning, New York, USA). For IGF‐1 treatment, cultures were differentiated in ventral neural induction media (VNIM) media 21 supplemented with recombinant human IGF‐1 (5 ng/ml, Sigma‐Aldrich, San Louis, USA) until day 37, then in basal knockout serum‐free media 14 with 10 ng/ml IGF‐1 until day 90 (Fig. 1Aii). Control cultures were differentiated in parallel in the absence of IGF‐1 (Fig. 1Ai). Time‐lapse capture of culture morphology was achieved using a BioStation CT (Nikon Corporation, Tokyo, Japan).

### Immunocytochemistry and TEM

EBs were fixed for more than days 30–90 of differentiation and immunocytochemistry performed on cryostat sections as previously described 21. Sections were reacted against a panel of retinal, lens, and corneal‐specific antibodies, listed in Supporting Information Table S1. The number of investigated structures per antibody varied slightly given the higher frequency of optic structures generated with IGF‐1; however, at least five structures from each differentiation time point across each experimental group were immunostained and this analysis was repeated for all the biological triplicates. Images were obtained using a Zeiss Axio Imager.Z1 microscope with ApoTome.2 accessory equipment and AxioVision software. For TEM, tissues were fixed in glutaraldehyde and processed by the Electron Microscopy Research Service at Newcastle University. The human embryonic and fetal material was provided by the Human Developmental Biology Resource (http://hdbr.org) under ethics permission (09/H0906/21). Due to the precious nature of this material, we restricted the use of this tissue to two sections per antibody.

### Electrophysiology

EBs were dark adapted for 24 hours prior to recording, then loaded with Fura2‐AM (10 µM, Molecular Probes/Life Technologies, Carlsbad, USA) for 45 minutes at 37°C (95% O_2_/5% CO_2_) followed by perfusion (1 ml/minute) with oxygenated artificial Cerebrospinal Fluid (aCSF; 118 mM NaCl, 25 mM NaHCO_3_, 1 mM NaH_2_PO_4_, 3 mM KCl, 1 mM MgCl_2_, 2 mM CaCl_2_, and 10 mM glucose) for 1 hour. Cells were exposed to 8‐br‐cGMP (cGMP, 1 mM, Sigma) in aCSF. Control recordings were performed with aCSF alone, 8‐br‐cGMP in CaCl2‐free aCSF supplemented with calcium channel blocker Cobalt (II) chloride (CoCl2, 4 mM, AnalaR) and 10 mM HEPES, or 8‐br‐cGMP in aCSF supplemented with glutamate blockers (DL‐AP5, 50 µm, Tocris (Bristol, UK); DL‐AP4, 20 µm, Tocris; CNQX, 10 µm, Tocris) to ensure that responses were not due to activity in second order neurons. Fluorescence was monitored using an inverted Olympus IX71 microscope and images (excitation 380 nm, 800 ms exposure) acquired using a Quantem 512SC camera (Photometrics) and MetaMorph Software (Molecular Devices). For each stimulation, 20 seconds of baseline activity was recorded first. Fluorescence change was analyzed using the formula % Δ*I*/*I*_o_ = (*I*_1_ − *I*_o_) × 100/*I*_o_, where *I*_o_ was the average cellular fluorescence during baseline activity and *I*_1_ following stimulation. Significance was determined using a Student's *t* test (*p* < .01). Retinal cells that showed a specific cGMP response were marked for their location and once the calcium imaging recording had taken place, immunocytochemistry analysis with photoreceptor‐specific antibodies (Recoverin, Opsin blue/red green, Rhodopsin, Supporting Information Table S1) was performed to confirm that the response was derived from photoreceptor cells.

### IGF‐1 Receptor Signaling Interference

hESCs were expanded and differentiated as described. For interference of IGF‐1 receptor signaling, an IGF‐IR‐specific pharmacological inhibitor ([7 µM], AG1024, Stratech, Newmarket, Suffolk) was added to differentiation media for the entire differentiation period. To block/neutralize IGF‐II receptor signaling, media were supplemented with goat IgG anti‐human IGF‐IIR antibody ([0.25 µg/ml], AF2447, R&D) throughout differentiation.

### Counts and Statistical Analysis

For each experimental condition, the total number of optic vesicles and optic cups were counted under an IVF hood dissection microscope on days 15, 30, 45, and 60 of differentiation. Data are presented as the mean ± SEM (*n* = 3). Statistical significance was determined using a two‐tailed Student's *t* test at 95% confidence (*p* > .05). Where a frequency was compared with zero (Figs. 1I, 5B), the probability was determined using a one sample *t* test or two‐tailed Student's *t* distribution, where *X* = mean/SEM.

## Results

### 2D Versus 3D Differentiation Culture and Development of the Optic Cup

In our previous work, we have shown that hESC/hiPSC‐derived embryoid bodies (EBs) differentiating under adherent 2D conditions form neural rosettes and optic vesicle‐like structures containing retinal progenitor cells which give rise to photoreceptor‐like cells and expanding sheets of RPE 21. However, the formation of complex structures that clearly resembled the optic cup was not obvious 21, most likely due to the physical constraints of 2D culture conditions. The culture of EBs in 3D suspension either throughout differentiation 24 or in the final stages 27 has been shown to be instrumental for generation of optic vesicle and optic cup structures from hESCs and hiPSCs. We combined the minimal medium shown in our previous publication 21 to generate photoreceptor progenitors and RPE (Fig. 1Ai) with 3D culture conditions throughout the differentiation process and observed the development of phase bright tissue at the edge of EBs reminiscent of the evaginating optic vesicle (Fig. 1B) and invaginating optic cup (Fig. 1C, 1D). We classified this minimal differentiation group as the “control.” A small proportion of control EBs gave rise to expanding sheets of cells which arose bilaterally and then underwent folding to become more convoluted over time (Supporting Information Movie S1).

Developmental studies have shown that across the entire spectrum of signaling molecules required for eye specification in any model system, injection of IGF‐1 in Xenopus embryos specifically promotes the formation of ectopic eyes containing multilayered neural retina, RPE, and sometimes lens 29. IGF‐1 is largely produced by the liver, although a number of other tissues including those of the central nervous system can produce IGF‐1 locally. IGF‐1 expression in postnatal rat retina is higher than that seen in the adult, consistent with a more prominent role during early developmental stages 30. Most importantly, expression of the IGF‐1 receptor is observed in the very early stages of optic cup formation in 28–32 days old human embryos and is subsequently restricted to the lens and RPE by 6 weeks of development 31. To assess the role of IGF‐1 on the efficiency of optic vesicle and cup formation from hESC, we supplemented our cultures with 5 and 10 ng/ml IGF‐1 at different stages of differentiation as shown in Figure 1Aii. We named this experimental group IGF‐1 (Fig. 1E–1G). We noted that addition of IGF‐1 led to a significant increase (*p* = .026) in the formation of optic vesicles during the first 15 days of the differentiation process (Fig. 1H). While no optic cup‐like structures were observed at day 45 of differentiation in the control group, these were clearly present in the IGF‐1 group (Fig. 1I) and continued to develop and increase in number giving rise to a 4.3‐fold increase in the IGF‐1‐treated group compared to controls. In parallel, other growth factors and signaling agonists/antagonists 21 were tested under the 3D culture conditions (Noggin, Dkk‐1, Lefty A, Shh, and T3); however, none of those resulted in an obvious increase in formation of optic vesicles or optic cups when compared with the control group (data not shown). qRT‐PCR analysis did not reveal significant changes in expression of pluripotency and neuroectodermal marker *SOX2* or cell cycle regulator *CYCLIN D1* (Supporting Information Fig. S1); however, IGF‐1 addition to the differentiation media caused a significant increase in the expression of eyefield marker *PAX6* and retinal progenitor marker *CHX10*. Together these data suggest that IGF‐1 enhances retinal differentiation through regulating formation of eyefield and retinal progenitor emergence rather than enhancing proliferation of early progenitors; this however remains to be investigated further and is currently ongoing in our group.

**Figure 2:**
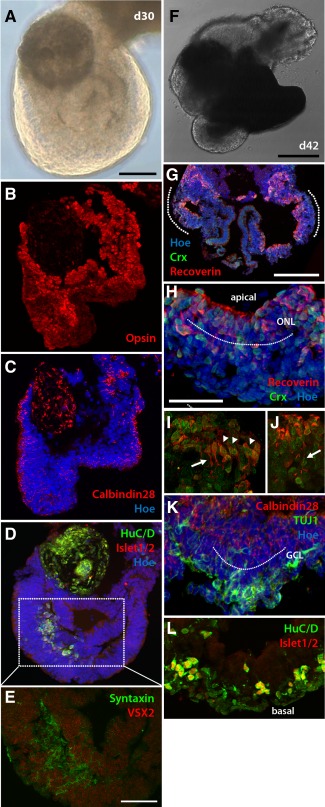
A small number of floating embryoid bodies differentiating under control conditions can yield primitive optic cup‐like structures containing multiple retinal phenotypes. **(A–E):** The expression of photoreceptor red‐green opsin (B), Calbindin 28 (C), HuC/D, and Islet1/2 (D) indicated the emergence of retinal phenotypes as early as day 30; however, mature morphology was not observed at this stage. (E): The image shown here represents the tissue section adjacent to the one shown in D which was processed by immunohistochemistry for the expression of Syntaxin and Vsx2. Enlargement of the equivalent areas [represented by the dotted line in (D) and image shown in E] suggests colocalization of Syntaxin staining with HuC/D and Islet1/2 immunopositive cells, indicating the presence of amacrine cells and the formation of a primitive inner plexiform layer. **(F–L):** An example of clearly identifiable developing retinal tissue arising under control conditions in vitro on day 42 of differentiation (F), which, upon sectioning after 90 days of differentiation (G–L), revealed a region displaying bilateral retinal development (G). Retinal tissue contained apically positioned photoreceptors (H–J) extending axons (white arrows, panels I, J) and inner segments (white arrowheads, panel I), and basally located ganglion cells (K, L) within the putative ONL and GCL layers, respectively (both depicted by the dotted lines) with medially placed amacrine cells (L). (G–L): Sections through the region demarcated by the dotted line in (F). Scale bars: A = 100 µm, E, H = 50 µm, F, G = 200 µm. Abbreviations: GCL, ganglion cell layer; ONL, outer nuclear layer.

Furthermore, we investigated the internal composition of these optic cup‐like structures arising from floating EBs in the control and IGF‐1 treatment groups. Under control conditions on day 30, we observed the presence of layered retinal neuroepithelium containing cells that were immunopositive for photoreceptor, amacrine and ganglion cell markers (Fig. 2A–2E). Colocalization of the presynaptic marker Syntaxin with HuC/D (staining amacrine and ganglion cells) and Islet1/2 (a ganglion cell marker) suggested that a putative inner plexiform layer was starting to form (Fig. 2D, 2E); however, no mature morphology was observed and laminar organization was variable between samples, with most sampled optic cups containing a reverse laminar organization (i.e., photoreceptors on the basal aspect [Fig. 2B]). A very small number (only 4% ± 2%) of the optic cups sampled from control conditions achieved more typical laminar organization by day 90 (Fig. 2F–2L), with retinal tissue occasionally occurring bilaterally (Fig. 2G) and showing typical polarization with apically positioned photoreceptor cells (Fig. 2H–2J) and basally located ganglion and amacrine cells (Fig. 2K, 2L). At no time point during differentiation did photoreceptors emerging under control conditions show outer segments despite staining with mature markers such as Opsin and Rhodopsin. Folding and invagination of retinal neuroepithelium occurred in some, but not all examples; in fact many (Supporting Information Movie S2) went on to develop RPE and laminated retinal tissue without invagination taking place (Fig. 2F–2L).

**Figure 3:**
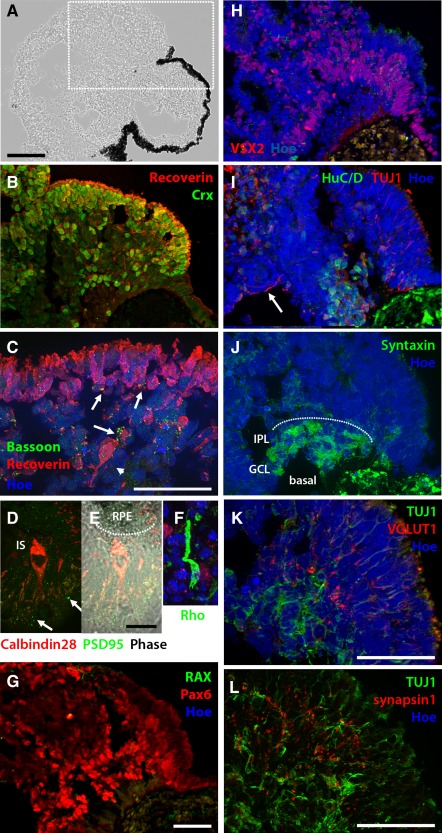
Treatment of differentiating cells with insulin‐like growth factor 1 orchestrated the formation of organized laminated neural retinal tissue containing multiple retinal phenotypes. A section through the optic cup shown in Figure 1E–1G showing its internal structure on day 90 of differentiation **(A)** and sister sections showing immunostaining of the area outlined by the white rectangle (**B**, **C** and **G–L**). Crx^+^ (B), and Recoverin (B, C) immunopositive cells were found on the apical side of developing retinal tissue. (C): Recoverin immunopositive photoreceptors typically formed a radially arranged apical outer nuclear layer (ONL) and expressed Bassoon at their terminals (white arrows). **(D–F):** Developing photoreceptors expressed mature markers and exhibited morphology typical of maturing photoreceptors including the emergence of inner‐ and outer segment‐like structures. Furthermore, human embryonic stem cell‐derived photoreceptors displayed an ellipsoid shaped distal portion of their IS (D), a morphological feature equivalent to 24–25 weeks of gestation, that colocalized in the correct orientation with RPE cells (E) and expressed PSD95 on their axon terminals (white arrows, panel E) indicating synaptic formation with adjacent cells. (G): Pax6^+^ cells were situated toward the basal aspect and (H) VSX2 medially. (I): Inner retinal neurons colocalized with syntaxin staining (J) indicating the region of the future GCL and IPL (indicated by the dotted line in panel J). (K, L): Punctate expression pattern of synaptic markers VGLUT1 and Synapsin 1 juxtaposed to the basal aspect of the developing ONL and inner TUJ1+ neuronal processes indicating formation of synaptic vesicles and conventional synapses between photoreceptors, second‐ and third‐order retinal neurons. Scale bars: A = 100 µm, C, G, K, L = 50 µm, E = 20 µm. Abbreviations: GCL, ganglion cell layer; IPL, inner plexiform layer; IS, inner segment; RPE, retinal pigmented epithelium.

Immunostaining of IGF‐1‐treated EBs on day 90 revealed structures similar to the developing neural retina located adjacent to developing RPE (Fig. 3A, Supporting Information Fig. S1). The majority (>60%) of sampled IGF‐1‐treated optic cups displayed laminar organization, with an outer layer of Crx and Recoverin immunopositive photoreceptor cells (Fig. 3B) and a medial band of retinal progenitors (Fig. 3G, 3H; the development of this IGF‐1‐treated specimen over time in culture can be seen in Supporting Information Movie S3). While Crx^+^ cells could be observed throughout developing retinal neuroepithelium (Supporting Information Fig. S2D), these became apically positioned along with the onset of Recoverin expression (Fig. 3B, 3C). While we have previously observed that photoreceptors emerging under adherent 2D conditions do not survive over long‐term culture 21, those differentiating in 3D in the presence of IGF‐1 survived and demonstrated further maturation for up to 90 days, the latest time point tested. Photoreceptor cells typically aligned side‐by‐side to form a radially arranged apical layer (Fig. 3B, 3C), expressing Bassoon on their terminals, suggesting the formation of ribbon synapses (Fig. 3C, arrows) and PSD95 at their basal surface (Fig. 3E). Photoreceptor cells exhibited inner and outer segment‐like structures following immunostaining for mature photoreceptor markers (Fig. 3D–3F). Interestingly, these photoreceptor inner‐ and outer‐like segments were observed as early as day 45 of differentiation in the IGF‐1‐treated group and were located adjacent to developing RPE (Fig. 3E). Inner retinal neurons were identified toward the basal surface and developing ganglion cells projected their axons along the developing nerve fiber layer (Fig. 3I, arrow and Supporting Information Fig. 2G). Syntaxin staining confirmed the presence of a developing inner plexiform layer (Fig. 3J). Furthermore, the expression of presynaptic marker, VGLUT1 which is essential for transmission of visual signals from photoreceptors to second‐ and third‐order neurons, was observed in a punctate pattern juxtaposed to the basal aspect of photoreceptors and the apical aspect of TUJ1‐positive inner retinal neuronal processes (Fig. 3K), indicative of the formation of synaptic vesicles. A similar punctate pattern of expression localized adjacent to TUJ1‐positive neuronal processes was also observed for Synapsin 1, suggestive of formation of conventional synapses between retinal ganglion cells and neurons of the inner nuclear layer (Fig. 3L).

By day 90, very few examples (<5%) of arising neural retina appeared immature, characterized by a large number of proliferating cells and retinal progenitors as detected by Ki67 and VSX2, respectively, (Supporting Information Fig. S2A–S2C); nonetheless the establishment of apical‐to‐basal polarization could be observed (Supporting Information Fig. S2E–S2G). While IGF‐1 treatment enhanced the formation of laminated neural retinal with apically placed photoreceptors (Supporting Information Fig. S3A), a minority of differentiating EBs contained internal cellular rosettes exhibiting a central hollow lumen surrounded by retinal phenotypes at varying stages of development. In such examples, Crx^+^ cells were found surrounding the lumen (inverted apical surface, Supporting Information Fig. S3B) and HuC/D^+^/TUJ1^+^ cells (putative ganglion cells) were found toward the periphery (inverted basal surface) of retinal rosettes (Supporting Information Fig. S3C), akin to results previously reported 32. Even in inverted form, developing photoreceptors expressed photoreceptor markers (Rhodopsin and Recoverin) toward the lumen and Bassoon at their terminals (Supporting Information Fig. S3B, S3D–S3F). A similar number of rosettes were also seen under control conditions, but they did not express mature photoreceptor markers, nor Bassoon or Synaptophysin (data not shown). Together, these results indicate that IGF‐1 acts to enhance the formation of laminated neural retina featuring multiple, well differentiated retinal phenotypes which reside in their native laminar orientation, form synapses with each other and morphologically develop to greater maturity than those generated in the absence of IGF‐1.

### IGF‐1 Pathway Signaling Plays a Key Role in Optic Formation and Survival

**Figure 4:**
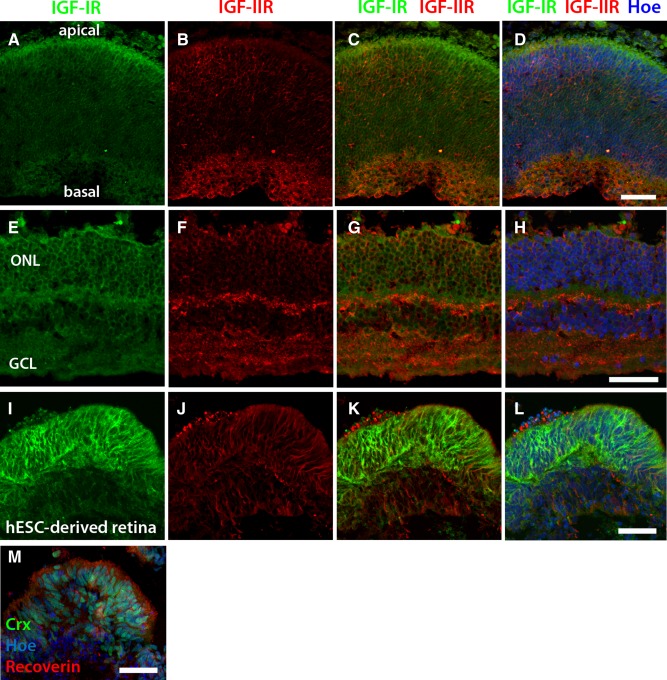
IGF pathway receptor expression in human eye tissue and retinal tissue derived from hESCs. **(A–H):** The expression of IGF‐IR and IGF‐IIR in (A–D) human fetal retina at 12 weeks of development and (E–H) adult human retina. IGF‐IR expression was visualized at the apical surface of the developing retina, while its expression in the adult retina was localized to photoreceptors in the ONL. IGF‐IIR clearly localized to the basal aspect of the neuroblastic layer in developing retina and the GCL, IPL, and OPL in adult retina. **(I–M):** In line with our observations from human retina (A–H), IGF‐IR staining was associated with regions of hESC‐derived photoreceptors, while IGF‐IIR staining also labeled the inner retina. Scale bars: D, H, L, M = 50 µm. Abbreviations: GCL, ganglion cell layer; hESC, human embryonic stem cell; IGF, insulin‐like growth factor; ONL, outer nuclear layer.

Retinal photoreceptors express IGF‐1 receptors (IGF‐IR) 29. Our studies of IGF‐IR and IGF‐IIR expression in the human retina show that IGF‐IR expression is localized to the apical border of the outer neuroblastic layer in fetal retina (Fig. 4A, 4C, 4D) and is strongly associated with the outer nuclear layer in adult retina (Fig. 4E, 4G, 4H), while IGF‐IIR expression is mainly observed in the inner neuroblastic layer in fetal retina but with weak widespread expression across the developing retina, and in the retinal ganglion cells, inner and outer plexiform layers of the adult retina (Fig. 4B, 4D, 4F, 4H). In line with these observations, our analysis of differentiating EBs confirmed that IGF‐IR staining was restricted to developing photoreceptors of the outer neuroblastic layer (Fig. 4I, 4K–4M), with IGF‐IIR expression weaker by comparison and more widespread across developing retinal tissue (Fig. 4J–4L and a sister section showing the location of developing photoreceptors in Fig. 4M).

**Figure 5:**
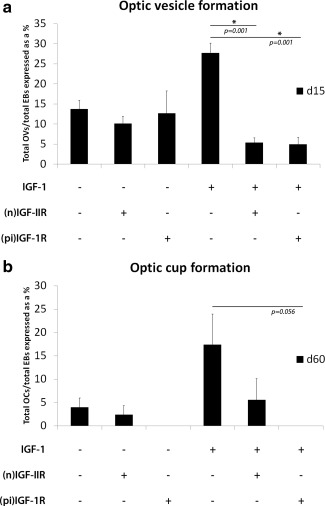
Interference of IGF‐1 pathway signaling significantly inhibits optic vesicle and optic cup formation. **(A, B):** Treatment of differentiating EBs with IGF‐1 resulted in a significant increase in optic vesicle production (A) and higher average frequency of optic cup formation (B). Pharmacological inhibition of the IGF‐IR (pi IGF‐IR) significantly reduced the formation of optic vesicles in the presence of exogenous IGF‐1 (A) and did not permit the survival of EBs past 50 days of differentiation both in the presence and absence of exogenous IGF‐1 (B). Neutralization of the IGF‐IIR (n)IGF‐IIR significantly reduced optic vesicle formation by day 15 in the presence of exogenous IGF‐1 (A) but did not replicate the inhibitory effects upon optic cup survival seen with IGF‐IR inhibition (B), implicating the IGF‐1/IGF‐IR signaling pathway as an important mediator of retinal development and survival. * indicates statistical significance, *p* < .05 as indicated. Abbreviations: EB, embryoid body; IGF, insulin‐like growth factor.

The 3D differentiation of hESC cultures in the presence of IGF‐1 gave rise to a significant increase (*p* = .026) in the number of observed optic vesicles compared with control cultures by day 15 (Fig. 5A). To confirm the contribution of the IGF‐1 signaling pathway in this process, we differentiated cells in the presence of a pharmacological inhibitor of the IGF‐IR [pi(IR)] or a neutralizing antibody directed against the IGF‐II receptor, IGF‐IIR ((n)IIR), in the presence (+) or absence (−) of IGF‐1 supplementation (Fig. 5A, 5B). Interference of both IGF‐IR and IGF‐IIR signaling significantly reduced optic vesicle production from differentiating EBs at day 15 in the IGF‐1 supplemented groups (*p* = .001) but not in the control groups, indicating an important role for exogenous IGF‐1/IGF‐IR/IGF‐IIR signaling in this first important window of differentiation (Fig. 5A). Inhibition of IGF‐IR signaling also interfered with optic cup formation as these were undetectable at day 60 in both the control and IGF‐1 treatment groups (Fig. 5B), while the IGF‐IIR inhibited group remained capable of developing a reduced number of optic cups, suggesting an important role for both endogenous and exogenous IGF‐1/IGF‐IR‐mediated signaling in optic cup development. Together these data indicate that endogenous and exogenous IGF‐1 signaling mediated through IGF‐IR/IGF‐IIR play an important role in formation of optic vesicle and optic cup formation as well as their continued survival in culture.

### Transmission Electron Microscopy Analysis

**Figure 6:**
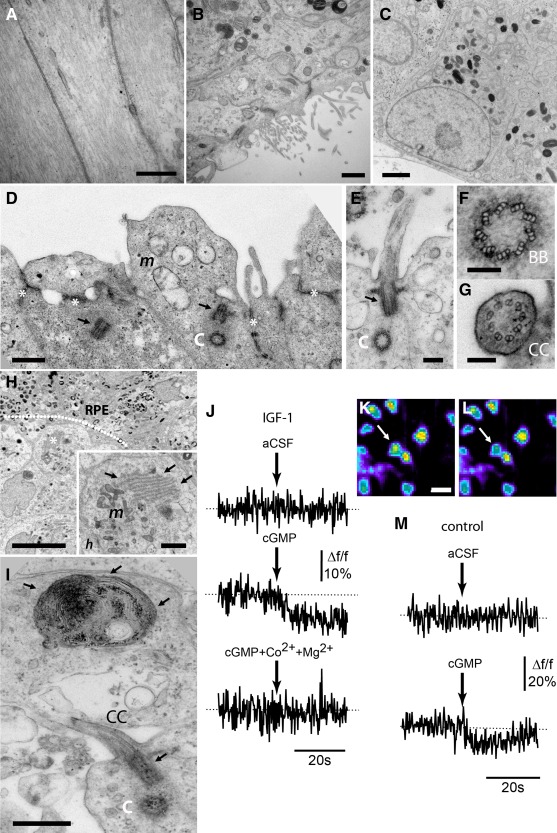
Transmission electron microscopy analysis of human embryonic stem cell (hESC)‐derived cell types and electrophysiological analysis of photoreceptors. **(A–I):** Ultrastructural analysis of differentiating hESCs treated with IGF‐1. (A): Optic cup‐like structures contained neural cells with prominent neurofilament and (B, C) pigmented cells which expressed cilia and (B) showed polarization of melanosomes (C). (D): An outer limiting membrane was observed (white asterisks) alongside developing photoreceptor cells which exhibited mitochondrion‐rich (*m*) inner segment‐like membrane protrusions adjacent to photoreceptor‐specific microtubule arrangements (D–G), revealing the presence of the photoreceptor centriole, “C” (D, E), a clearly identifiable 9 × 3 + 0 basal body complex, “BB” (D–F, I) and 9 × 2 + 0 connecting cilium, “CC” (E, G, I). (H, I): The examination of photoreceptor cells developing adjacent to a layer of RPE (demarcated by the white dotted line) revealed ultrastructural features (H, asterisk and *h*, I arrows) similar to nascent outer segment discs that were found in proximity to mitochondrion (*m*). (I): These structures were present alongside a single cellular centriole (C), basal body complex (arrow) and connecting cilium (CC), indicating the development of the photoreceptor connecting cilium. **(J, M):** Raw traces of cGMP‐induced fluorescence changes in individual cells labeled with Fura2‐AM, expressed as percentage change from baseline fluorescence in IGF‐1 treated (J) and control cells (M) on day 45 of differentiation. The traces show changes in fluorescence when exposed to a 50 µl aCSF puff (top trace) and cGMP puff delivered at the time indicated by the arrow. In photoreceptor cells cGMP triggers an increase in calcium influx, reflected by a decrease in fluorescence, whereas aCSF has no effect. The fluorescence images **(K, L)** to the right of (I) illustrate the same cell (indicated by a white arrow) together with surrounding cells in the culture represented in false fluorescence colours before (averaged during 10 seconds; top image) and during (averaged during 20 seconds; bottom image) cGMP application. (J): The calcium response to cGMP (middle trace) disappears in the presence of cobalt chloride (2 mM) and magnesium chloride (3 mM) (and no calcium chloride) in the perfusate (lower trace). Scale bars: A, D, I = 500 nm, B = 1 µm, C,*h* = 2 µm, E = 200 nm, F, G = 100 nm, H, K = 10 µm. Abbreviation: RPE, retinal pigmented epithelium.

Transmission electron microscopy (TEM) analysis of differentiating cells (Fig. 6A–6I) revealed a high proportion of neurofilament‐containing cells and pigmented cells which featured cilia, some of which demonstrated polarized location of melanosomes (Fig. 6C) indicating maturing RPE cells. A layer of adherens junctions demarcating the position of the outer limiting membrane was observed (Fig. 6D, asterisks) alongside developing photoreceptor cells which exhibited mitochondrion‐rich (Fig. 6D, *m*) inner segment‐like cell membrane protrusions and photoreceptor‐specific microtubule presentations (Fig. 6D–6G), confirming the presence of the photoreceptor centriole (Fig. 6D, 6E), basal body complex (Fig. 6D–6F, 6I; a 9 × 3 + 0 arrangement), and connecting cilium (Fig. 6E, 6G, 6I; a 9 × 2 + 0 arrangement). The ultrastructural examination of photoreceptor cells developing adjacent to RPE revealed the presence of organized structures which may represent nascent outer segment discs (Fig. 6H, 6I), similar to that shown in the study by Zhong et al. 27. The definitive identification of photoreceptor outer segments becomes much easier once the morphologically identifiable tall stack of outer segment discs has clearly formed. In our study, as in 27, the outer segment‐like structures which have been identified are comparatively small organized stacks, which may represent the early emerging photoreceptor outer segment following the production of a minimal number of outer segment discs. Although it is difficult to distinguish early photoreceptor outer segments from other well‐organized organelles, the topological proximity of these structures to the RPE (Fig. 6H) and their association with the presence of a single cellular centriole and connecting cilium (Fig. 6I), however, strengthens this notion, and indicates that photoreceptor cells derived from stem cells may demonstrate some capacity for outer segment disc formation and assembly. Importantly, these nascent outer segment disk‐like structures (Fig. 6H/*h*, 6I) could be observed in the IGF‐1‐treated group only.

### Functional Studies

In order to determine whether developing photoreceptors contained the machinery necessary for phototransduction, we plated differentiating EBs onto poly(l‐ornithine) and laminin‐coated six‐well plates for 15 days prior to optical recording using calcium imaging. Membrane disks in the outer segment of native photoreceptors contain a photopigment (e.g., the rod‐specific pigment rhodopsin consists of the protein opsin and its cofactor retinal), and the cell membrane in outer segment contains cyclic nucleotide‐gated (CNG) cationic (sodium and calcium) channels. In the dark, cyclic guanosine monophosphate (cGMP) levels are high and thus photoreceptors are maintained in a depolarized state via the influx of Na^+^ and Ca^2+^ through cGMP‐mediated activation of CNG channels, known as the inward dark current. Exposure to light causes retinal to isomerize to trans‐retinal, leading to activation of multiple G‐proteins and phosphodiesterase 6 which, in turn, degrades cGMP, resulting in CNG channel closure and membrane hyperpolarization. We investigated the functionality of CNG channels (representing the last step in the phototransduction cascade) in our hESC‐derived photoreceptors. On days 30, 45, and 90 of differentiation, cells were exposed to a membrane‐permeable cGMP analogue (8‐br‐cGMP). As 8‐br‐cGMP opens the cationic channel associated with phototransduction, it triggers a Ca^2+^ influx in photoreceptors reminiscent of the inward dark current. Loading cells with the fluorescent Ca^2+^ indicator Fura‐2 therefore enables the visualization of changes in intracellular calcium associated with the opening or closing of CNG channels, with a decrease in Fura‐2 fluorescence emission representing an increase in calcium influx.

When exposed to 8‐br‐cGMP, IGF‐1‐treated cultures showed decreased fluorescence (Fig. 6J–6L), indicating a cGMP‐mediated increase in Ca^2+^ influx. This effect was abolished when cGMP was delivered alongside the calcium channel blockers cobalt chloride and magnesium chloride in the perfusate (Fig. 6J, bottom panel). On day 45 of differentiation 24.1% (±7.8) of IGF‐1‐treated cells (*n* = 4; 587 cells analyzed) responded to cGMP, and this increased to 45.3% (±13.2) by day 90 (*n* = 4; 215 cells analyzed). These data corroborate our earlier observations on the timing of inner and outer segment‐like structure development (Figs. 3D–3F, 6D–6I) and support our observations that the supplementation of hESCs with IGF‐1 leads to the generation of photoreceptor cells, which exhibit features of advanced maturation as early as 6.5 weeks of the differentiation process.

### Formation of Accessory Eye Structures in the Presence of IGF‐1

**Figure 7:**
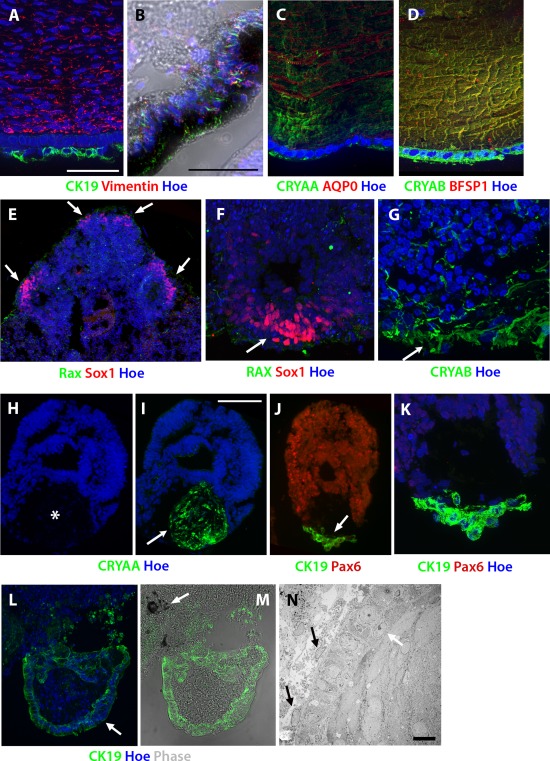
Insulin‐like growth factor 1 treatment facilitates the emergence of ocular accessory structures alongside retinal tissues. **(A–D):** Sections through human fetal cornea (A), pigmented ciliary margin (B), and lens (C, D) at 12 weeks of development stained for lens (CRYAA, CRYAB, γ‐crystallin, AQP0, and BFSP1) and corneal epithelial and stromal markers (CK19 and vimentin). The pigmented ciliary margin (panel B) was also immunopositive for vimentin and CK19. **(E, F):** Superficial regions of Sox1 expression were often observed adjacent to developing retinal tissue close to the surface of the embryoid body and this often occurred bilaterally (E, arrows). Superficial Sox1 expression (F) was accompanied by adjacent CRYAB immunostaining **(G)**, indicating the emergence of the lens vesicle at the exposed side. **(H–K):** A region of cells lacking nuclei (white asterisk) immunopositive for the lens marker CRYAA was observed between developing retinal tissue marked by PAX6 expression (J) and corneal epithelium immunostained for CK19 (J, K). **(L, M):** CK19 immunopositive regions did not overlap with emerging RPE as shown by the white arrow in M; **(N)** transmission electron microscopy analysis revealed structures resembling developing corneal tissue comprising of deeper corneal layers contained by a band of basal cells (white arrows) and surrounded by superficial cells (black arrows). Scale bars: A, B, K = 50 µm, J = 100 µm, L = 10 µm.

We also observed that regions of differentiating cells adjacent to developing RPE and retinal tissue were immunopositive for markers of accessory structures of the eye (Fig. 7). Sox1 is expressed in the lens vesicle shortly after the ectoderm gains a lens‐forming bias, but is not found in the neural retina 33, 34, 35. Sox1 is also coexpressed with RAX in the neural epithelium of the adjacent diencephalon 36. To confirm the specificity of corneal and lens antibodies we first performed immunocytochemical analysis on sections of developing human fetal eyes (Fig. 7A–7D) which showed specific expression patterns in the corneal epithelium and lens. For example, CK19 (known as marker of corneal epithelial progenitor) expression was found adjacent to Vimentin positive stromal region indicating development of corneal epithelium residing on corneal fibroblasts (Fig. 7A). We used the same antibodies to screen sections of EBs obtained from the IGF‐1 and control groups and observed that in IGF‐1 supplemented cultures, the portion of the neural rosette found toward the surface of an EB was often RAX negative and Sox1 positive (Fig. 7E, 7F), indicating a lens forming bias in these cells. This superficial region of RAX^−^/Sox1^+^ cells was observed only in IGF‐1‐treated cultures, and was often observed to arise bilaterally across EBs (Fig. 7E, arrows). Consolidating this result, we found superficial expression of lens‐specific Crystallin alphaB alongside RAX^−^/Sox1^+^ regions (Fig. 7G), indicating that the signals necessary for formation of the early lens were present in differentiating hESCs treated with IGF‐1 and that this was associated with retinal tissue formation. We also observed lens‐specific Crystallin alpha‐A (CRYAA) immunopositive regions of tissue which contained fewer cell nuclei than surrounding regions (Fig. 7H, 7I, asterisk), indicating the enucleation of lens cells similar to that which occurs during development. This CRYAA immunopositive region was also located next to CK19 stained area (Fig. 7J, 7K), highly suggestive of a developing corneal epithelium on the surface of EBs. In addition, corneal epithelial‐like structures were observed in TEM micrographs (Fig. 7N) where a core of cells forming a corneal‐like concentric pattern, encased by a band of basal cells (white arrows) and surrounded by superficial cells (black arrows) were found. Although CK19 has been suggested to stain RPE and lens during development, our experimental observations suggested that this was not the case (refer to Fig. 7L, 7M, where clearly the CK19 stained region on the outside of EB is not overlapping with the RPE location indicated by a white arrow in panel Fig. 7M) for RPE and lens structures arising during hESC differentiation. We can therefore conclude that in the presence of IGF‐1 additional ocular structures resembling corneal epithelial cells and the lens vesicle can arise in vitro alongside the developing neural retina.

## Discussion

The generation of mature photoreceptors from human pluripotent stem cells using simple and clinically compatible protocols that can be easily adapted by many labs is an important goal for clinical cell‐based therapies in the eye. To date, it has been shown that the differentiation of human pluripotent stem cells under 3D culture 24, 27, 32 in media supplemented with a handful of exogenous factors 24, 27 or without any factors 32 can result in the generation of optic cup structures. Yet only one very recent study 27 which involves multiple stages including the manual dissection of neural retina structures arising under 2D culture and the transfer of these to 3D culture conditions, alongside several stages of media supplementation including fetal bovine serum and taurine, has reported the generation of photoreceptors with features of maturation such as short stacks of outer segment‐like discs and phototransduction capacity after a culture period of 22–28 weeks. In this study, we report the development of a very simple method which uses 3D culture conditions throughout differentiation and involves the addition of one single exogenous factor, IGF‐1, to induce the differentiation of hESC to laminated neural retina containing photoreceptors displaying features of photoreceptor maturation as early as 6.5 weeks of differentiation. Furthermore, our differentiation method also results in the development of additional accessory ocular structures (cornea and lens epithelium) which, to the best of our knowledge, has not been demonstrated previously in recent 3D studies describing formation of laminated neural retina from hESC/hiPSC 24, 27.

During normal human development, the presence of synaptophysin in cone photoreceptor processes is visible around 12 weeks and is present in photoreceptor terminals by 16–17 weeks of gestation 37. Cones are arranged into mosaics by 18–19 weeks of gestation and by 24–25 weeks exhibit an ellipsoid distal portion of their inner segment 38. Syntaxin staining of the inner plexiform layer and nerve fiber layer is clear by 12 weeks of gestation and is present in amacrine and ganglion cells by 16–17 weeks. Taking this information from normal human development into account and linking this to what we have observed in our cultures, the stage of maturation of the retinal tissue derived under IGF‐1 treatment that we have observed suggests they correspond to a gestational age of 17–25 weeks 34, 38. This is surprising, given that we observe the first appearance of photoreceptors with inner and outer segment‐like structures as early as 6.5 weeks of our differentiation protocol, thus suggesting that certain aspects of normal development can be accelerated in vitro using key exogenous factors such as IGF‐1. Furthermore, our differentiation protocol is faster when compared with the recent study which showed that generation of maturing photoreceptors was possible only after 22–28 weeks using a more complicated regime 27. Herein we describe a simpler, more rapid, and good manufacturing practice—compatible method that lacks fetal calf serum and results in photoreceptor generation within a shorter time frame and which morphologically mature and display membrane properties compatible with phototransduction.

We had previously reported the loss of photoreceptors from our 2D adherent cultures for more than days 45–60 of differentiation 21, yet our recent work has revealed that this is not the case for EBs maintained in 3D culture and that in the presence of IGF‐1 photoreceptor cells continue to arise and mature up to day 90 of differentiation, the latest time point studied. One possibility is that the radial alignment of photoreceptors within a stratified retina containing other secondary neurones, as observed in our IGF‐1‐treated cultures, can help to maintain viability by mimicking a native retinal microenvironment which is lacking in adherent 2D retinal differentiation protocols. However, this may not be the only determining factor, as other groups have reported that emerging retinal laminae within optic vesicular structures derived from hESC or hiPSC lose their integrity over time 22, 24. In contrast to those studies, we have observed that photoreceptors arising in our 3D cultures are maintained over long‐term culture and continued to mature, alongside other associated retinal cell types. This increased viability could be due to presence of IGF‐1 in our culture media, as interference with IGF‐1/IGF‐IR signaling under our experimental conditions led to the loss of viability of retinal structures as early as day 45 of differentiation in addition to a significant decrease in the number of optic vesicles forming during the first 30 days of differentiation. Together these data suggest that IGF‐1 is a critical player in the eye field for the induction of the eye cup from pluripotent stem cells and for the maintenance of viable photoreceptors within laminated neural retina emerging during 3D differentiation.

The role of IGF‐1 in this process is not entirely unexpected. Addition of IGF‐1 to the culture media of differentiating hESC has been shown to significantly increase the number of retinal progenitor cells 13; however, its effects on optic vesicle and optic cup formation until now had not been reported. As retinal differentiation proceeds in vivo, IGF‐1 expression is observed in postmitotic retinal precursors which are in the process of differentiating into cones as well as in the inner segments of photoreceptors, promoting cone, and rod survival 39. This is supported by studies in mice lacking insulin receptor substrate 2, an essential component of the IGF‐1 signaling cascade, which show almost complete loss of photoreceptors by 16 months of age 40. Furthermore, ectopic overexpression of IGF‐1 in the adult mouse retina has been shown to significantly improve the integration of primary mouse photoreceptor precursors following transplantation 41. IGF‐1 is also observed in rod outer segments and has the ability to phosphorylate rod transducin, indicating that IGF‐1 signaling may also be involved in light transduction 42. Our expression studies in human fetal and adult retina show a localized expression of IGF‐IR in the developing and mature outer nuclear layer; however, a more widespread pattern of IGF‐IIR in the plexiform and ganglion cell layers was observed, suggesting a more focal role for IGF‐IR in photoreceptor development and maintenance than IGF‐IIR signaling. The expression pattern of these two receptors also fits well with signaling interference studies which indicate that inhibition of IGF‐IR but not IGF‐IIR significantly reduces the formation of optic cups from hESC and abrogates the viability of emerging neural retina in our 3D differentiation system. More importantly, this occurs both in the presence and absence of IGF‐1, indicating that endogenous IGF‐1/IGF‐IR signaling is a key orchestrator in the formation of viable optic cups from hESC. As the name suggests, IGF‐1 shares similarity with insulin and one can assume that insulin (which is present in most culture media) can cause the same effects as IGF‐1 by binding to IGF‐IR. This is however unlikely as binding of IGF‐1 to IGF‐IR is 20‐fold higher when compared with insulin binding to the same receptor 43, 44. Furthermore, when both IGF‐1 and insulin receptors are present at high levels hybrid receptors are formed; however, IGF‐1 is the more potent activator of both hybrid receptors when compared with insulin, strongly suggesting that the enhanced differentiation observed in the presence of IGF‐1 is due to the addition of this growth factor rather than presence of insulin in the culture media.

The emergence of laminated retinal tissue in the IGF‐1‐treated 3D cultures is not proof of functionality for the resident photoreceptors and other retinal cell types developing therein. To ascertain cell functionality we investigated synapse formation and photo‐transduction capabilities. Our results demonstrate clear Bassoon, VGLUT1, syntaxin, and Synapsin 1 staining, indicating the formation of synapses and emergence of an inner and outer plexiform layer in our hESC‐derived neural retina, indicative of synaptic zones connecting photoreceptors with bipolar and ganglion cells. IGF‐1 may have enabled this to occur, as it is reported to activate molecules associated with synaptogenesis 45. The presence of other ocular‐associated tissues may also be a contributing factor in enabling the more advanced photoreceptor maturation and the development of the outer segment‐like structures which we observed in our study. The formation of photoreceptors demonstrating inner and outer‐like segments situated directly adjacent to retinal pigmented tissue using our IGF‐1 differentiation method further demonstrates the powerful ability of hESCs to form neural retina that is correctly topologically oriented. Importantly we also demonstrate that a proportion of hESC‐derived photoreceptor cells express CNG channels and are sensitive to cGMP stimulation in a similar manner to native photoreceptors as early as 45 days (6.5 weeks) of differentiation. Furthermore, the number of responsive photoreceptor cells continues to rise as differentiation proceeds toward 90 days which, together with the observation of primitive outer segment‐like structures and clear photoreceptor connecting cilium and inner segments, demonstrates that these cells are undergoing continued development toward a functional photoreceptor cell.

Although the expression of lens‐specific genes has been reported in two publications describing the differentiation of hESC using a feeder layer approach 46, 47, to the best of our knowledge we are not aware of any reports documenting the emergence of accessory ocular structures accompanying neural retinal development, such as primitive lens or corneal epithelium in 3D studies that result in formation of laminated retina 22, 24, 27. We however observe that in the presence of IGF‐1 such structures emerge alongside developing retina in our study, challenging paradigms of retinal development. While lens fiber cell formation is rarely seen in ectodermal explants not exposed to the optic vesicle, the neural retina and fibroblast growth factor can stimulate lens development, and the vitreous humor is known to contain IGF‐1 which has been shown to potentiate lens fiber differentiation by inducing pulses of crystalline gene expression in rat lens epithelial cells, indicating that this may have contributed to the formation of early lens that we observed in IGF‐1‐treated populations 48, 49. The emergence of neural retina from hESC both with and without nearby lens cells in our cultures is an interesting result, given the ongoing debate surrounding the requirement of the lens in neural retinal formation 50, 51, 52, 53. We also observed the presence of RAX immunopositive cells within folded neuroepithelial sheets of cells found adjacent to developing retina, indicating a ventral cortical fate within these convoluted structures, such as diencephalic neural tissue, found adjacent to the eye field during normal development. This implies that not only lens and cornea but also other tissues normally found nearby to the developing eye are arising in parallel in vitro. This fascinating result clearly deserves further investigation.

## Conclusions

In summary, our results suggest that 3D culture conditions result in development of optic cup‐like structures containing RPE and neural retinal tissue. The addition of IGF‐1 in the absence of any other exogenous growth factors increased the efficiency of optic cup‐like structure formation and achieved a high level of retinal lamination and viability accompanied by photoreceptor‐specific ultrastructural morphology and the formation of accessory eye structures. The simplicity of this IGF‐1 supplemented culture system and the ability to produce maturing photoreceptors within a fully laminated retina within a shorter time frame than previously reported make this an ideal system for future cell based replacement therapies as well as in vitro modeling of inherited retinal dystrophies.

## Author Contributions

CBM designed and performed research, analysed data, wrote the paper and approved final version of manuscript; JC performed research and analysed data, approved final version of manuscript; MK performed research and analysed data; ES performed research, contributed to paper writing and final approval of manuscript; KW analysed data and approved final manuscript; DHWS contributed to paper writing and final approval of manuscript and fund raising; ML designed and performed research, contributed to paper writing, final approval of manuscript and fund raising.

## Disclosure of Potential Conflicts of Interest

Authors disclose no potential conflict of interest.

## Supporting information

Supplementary Information

Supplementary Information

Supplementary Information

Supplementary Information

Supplementary Information

Supplementary Information

Supplementary Information
