# Supplementary figures and images for: IGF‐1 Signaling Plays an Important Role in the Formation of Three‐Dimensional Laminated Neural Retina and Other Ocular Structures From Human Embryonic Stem Cells
Source: Stem Cells. 2015 May 13;33(8):2416–30. doi: 10.1002/stem.2023 (PMC4691326; doi:10.1002/stem.2023)

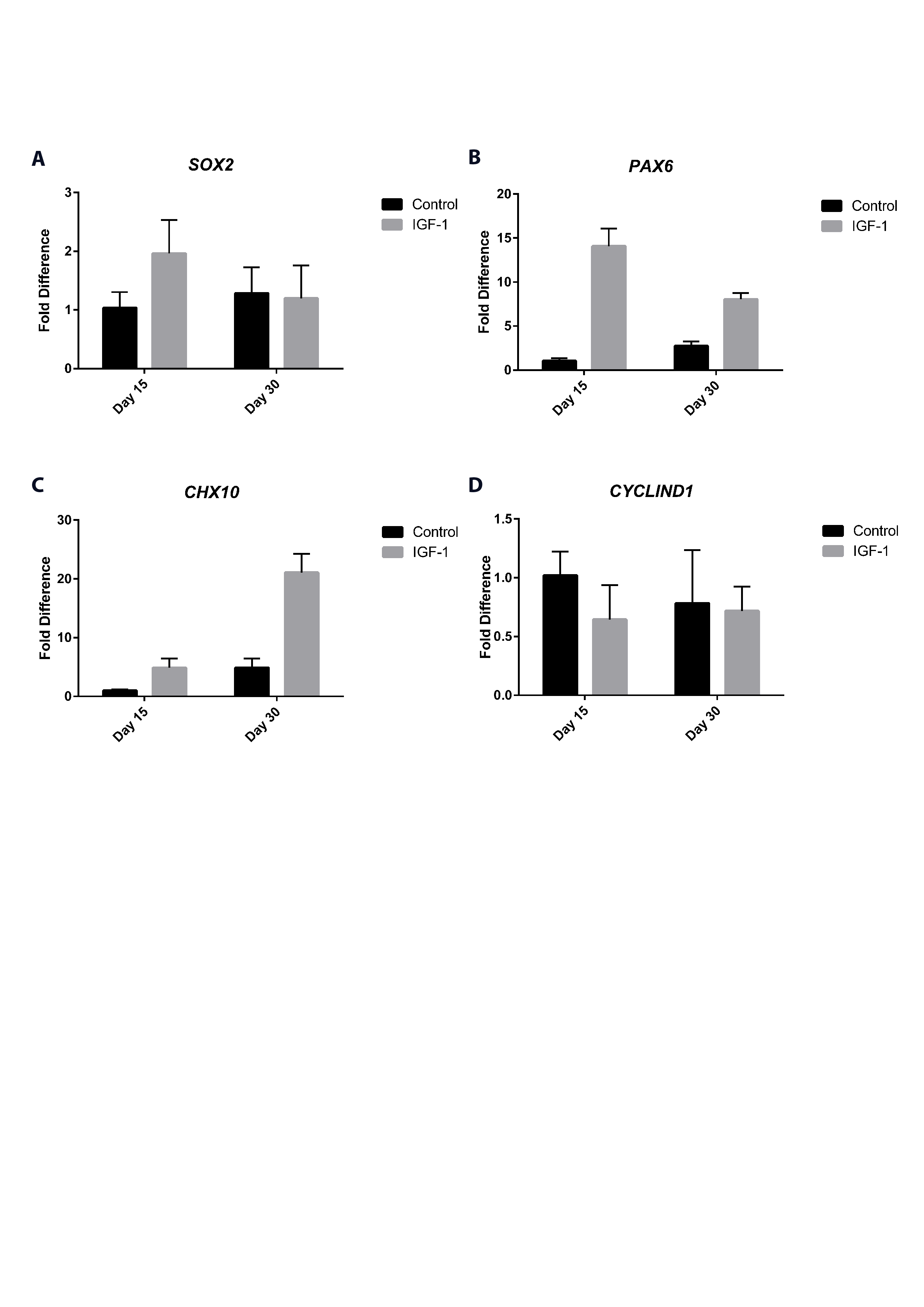

Supplement: Supplementary file 1 — Supplementary Information [file stem0033-2416-sd1.tif]

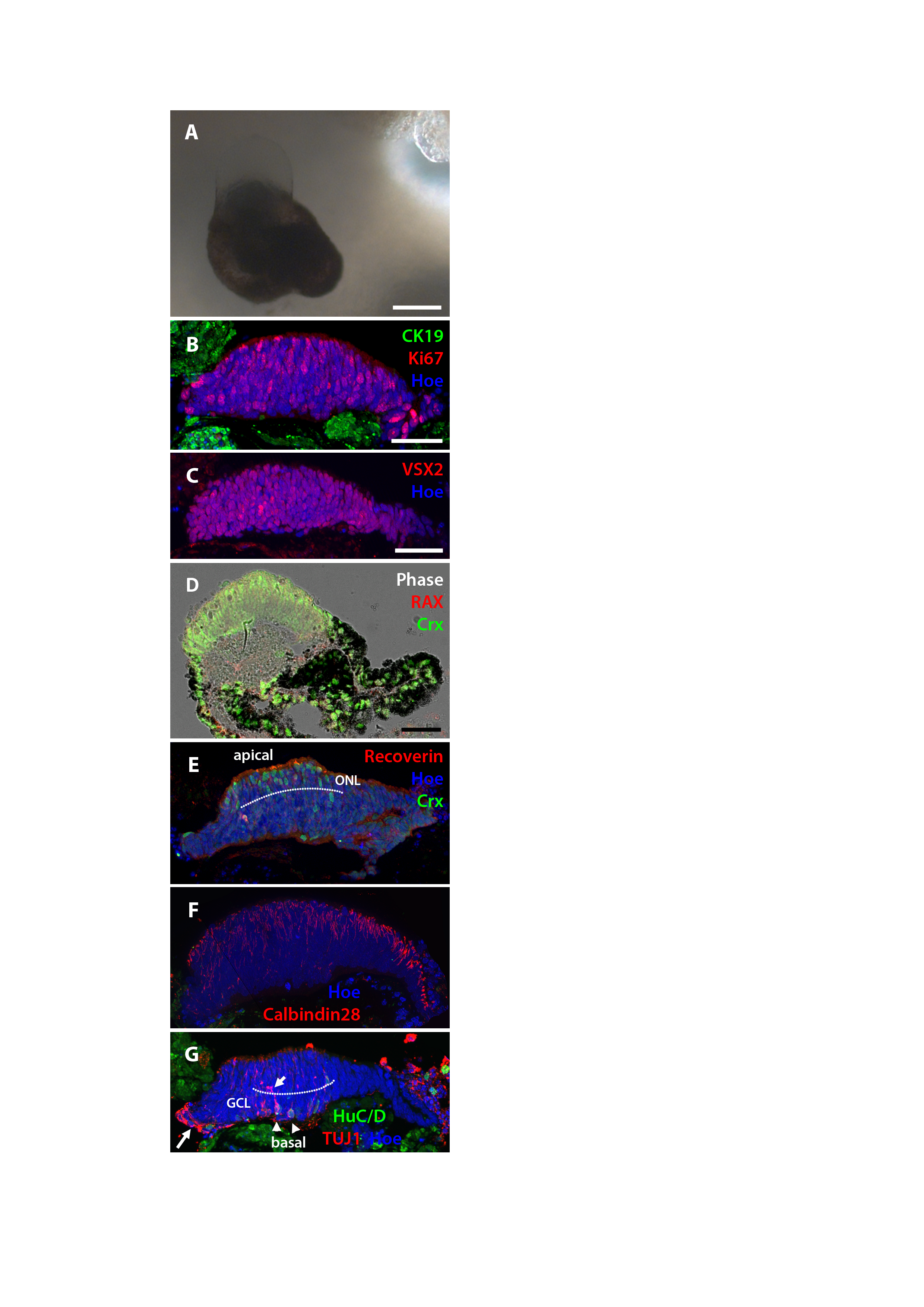

Supplement: Supplementary file 2 — Supplementary Information [file stem0033-2416-sd2.tif]

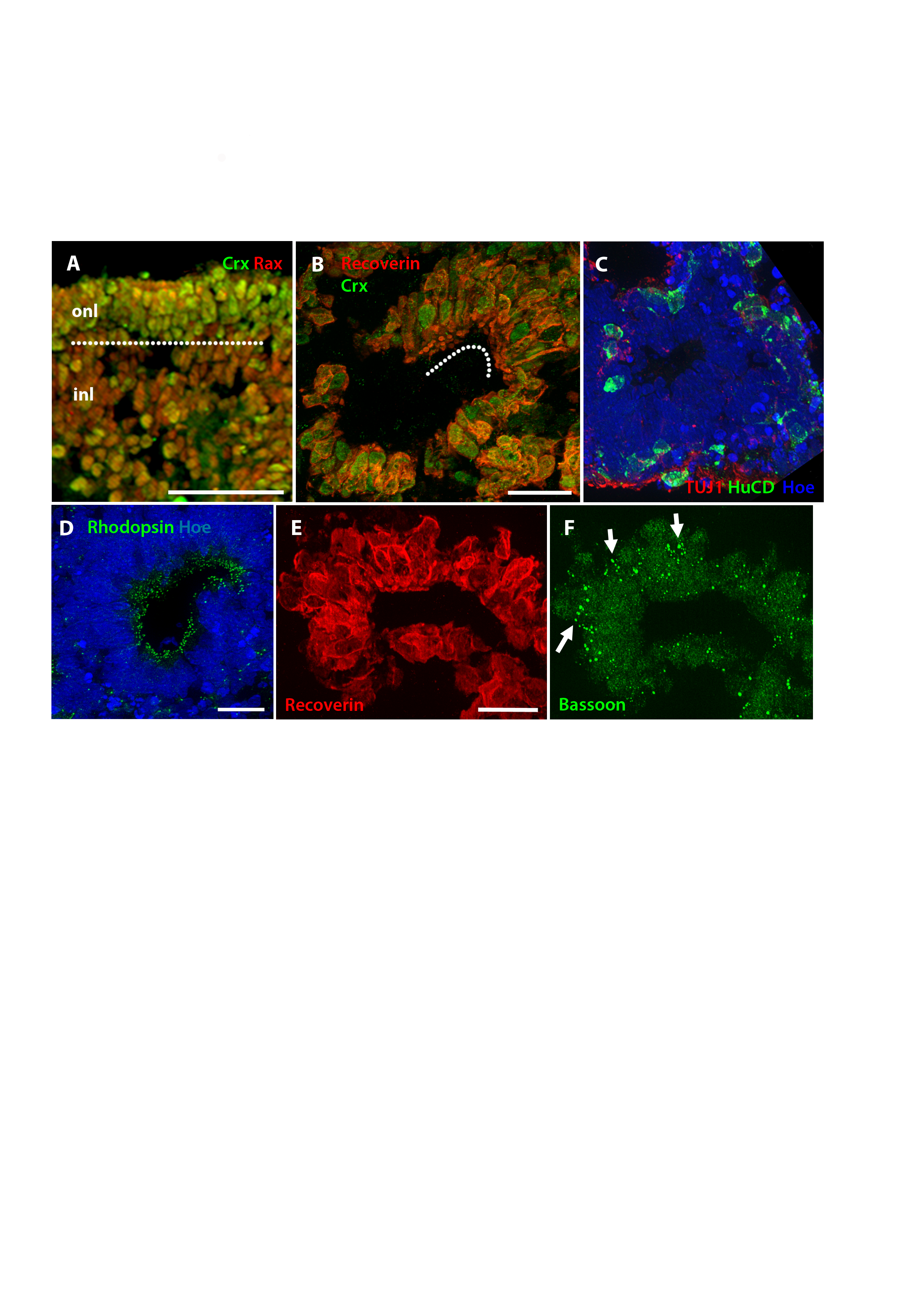

Supplement: Supplementary file 3 — Supplementary Information [file stem0033-2416-sd3.tif]
