# Supplementary material for: IGF‐1 Signaling Plays an Important Role in the Formation of Three‐Dimensional Laminated Neural Retina and Other Ocular Structures From Human Embryonic Stem Cells
Source: Stem Cells. 2015 May 13;33(8):2416–30. doi: 10.1002/stem.2023 (PMC4691326; doi:10.1002/stem.2023)
Supplement: Supplementary file 4 — Supplementary Information [file stem0033-2416-sd4.docx]

**Suppl. Table 1: Information on the antibodies used in this study.**

| **Antibody** | **Specificity** | **Dilution** | **Supplier** |
| --- | --- | --- | --- |
| anti-Bassoon | photoreceptor ribbon synapse, presynaptic terminals, co-localises with GABA and glutamate | 1:200 | StressGen PS003 |
| anti-Calbindin D-28K (Calbindin 28) | horizontal cells, cone photoreceptors (excluding S cones), On cone bipolar cells, wide field amacrine cells, large ganglion cells | 1:200 | Chemicon AB1778 |
| anti-Cytokeratin 19 (CK19) clone RCK108 | Peripheral corneal epithelium | 1:100 | Dako M088801-2 |
| anti-Crx | postmitotic photoreceptors | 1:200 | Abnova  H00001406-M02 |
| anti-Crystallin, alpha B (CRYAB) | lens epithelial cells | 1:5 | Sawada *et al*., 1993, 2D2B6 |
| anti-HuC/D | amacrine and ganglion cells | 1:200 | Molecular Probes A21271 |
| anti-Islet1/2 | retinal ganglion cells | 1:200 | Santa Cruz Biotechnology sc-30200 |
| anti-Ki67 | nuclear expression in proliferative cells during late G1, S, G2 and M phases of the cell cycle | 1:200 | AbCam Ab15580 |
| anti-Opsin blue | S cone photoreceptors | 1:200 | Millipore AB5407 |
| anti-Opsin red/green | L/M cone photoreceptors | 1:200 | Millipore AB5405 |
| anti-Opsin (clone RetP1) | N terminal of the rhodopsin molecule on rod photoreceptor cell bodies, inner and outer segments | 1:400 | Sigma-Aldrich O4886 |
| anti-Pax6 | neural progenitors, retinal progenitors | 1:300 | Covance PRB-278P |
| anti-Post Synaptic Density Protein 95 (PSD95) | membrane-associated guanylate kinase family synaptic protein, found in inner and outer plexiform layers | 1:200 | Millipore MAB1598 |
| anti-RAX  (clone 4F4) | retina and anterior neural fold homeobox; anterior neural fold, ventral diencephalon, optic vesicles, retinal progenitors | 1:100 | Sigma-Aldrich SAB1405061 |
| anti-RAX (against the N terminal) | retina and anterior neural fold homeobox | 1:200 | Aviva Systems Biology ARP31926 |
| anti-Recoverin | photoreceptors and midget cone bipolar cells | 1:300 | Chemicon AB5585 |
| anti-Rhodopsin | rod rhodopsin | 1:200 | Santa Cruz Biotechnology sc-57432 |
| anti-Sox1 | neuroectodermal tissue, lens vesicle and neural epithelium of adjacent diencephalon | 1:200 | Cell signalling technology 4194 |
| anti-Synaptophysin | presynaptic protein synaptic vesicles, expressed in photoreceptor terminals | 1:100 | Sigma-Aldrich S 5768 |
| anti-Syntaxin (clone HPC-1) | amacrine cell bodies and processes, inner plexiform layer | 1:200 | Sigma-Aldrich S 0664 |
| anti-Neuronal Class III β-Tubulin (TUJ1) | neurons, expressed in high levels in retinal ganglion cells | 1:800 | Covance MMS-435P |
| anti- Visual System Homeobox 2 (VSX2) | VSX2 protein, retinal progenitors and bipolar cells | 1:200 | Sigma-Atlas HPA003436 |
